# The Relationship between Childhood Trauma, Eating Disorders, and Sleep Quality among Registered Hospital Nurses in South Korea

**DOI:** 10.3390/healthcare8040490

**Published:** 2020-11-17

**Authors:** Gyehyun Jung, Jihyun Oh

**Affiliations:** 1 Department of Nursing, Jeonbuk Science College, Jeonbuk 56204, Korea; j500178@naver.com; 2 Department of Nursing, Daejeon University, Daejeon 34520, Korea

**Keywords:** eating disorders, childhood trauma, sleep quality, registered nurse

## Abstract

This cross-sectional study examined the relationship between childhood trauma, eating disorders, and sleep quality among registered hospital nurses in South Korea. Self-report questionnaires were answered by 279 nurses from six general hospitals. Factors affecting sleep quality were analyzed with a linear regression analysis. The factors that influenced sleep quality included age, alcohol consumption, chronic disease, BMI, and emotional abuse in childhood trauma. Hospital nurses need to increase their sleep health knowledge to maintain a healthy lifestyle while working as a nurse. Therefore, hospital and nursing managers should consider strategies to prevent and intervene in the sleep quality threats attributed to the adverse childhood experiences of hospital nurses.

## 1. Introduction

Nurses engage in shift work, leading to long-term disturbed sleep quality [1]. Others have reported that sleep disturbances among nurses are common, and are associated with memory loss, irritability, increased fatigue, and overeating [2,3,4]. Nurses working different shifts, including the night shift, are susceptible to poor sleep. Some rotational-shift nurses suffer from poor sleep quality. Compared to day and fixed shift nurses, over half of rotational shift nurses reported experiencing sleep problems [5,6]. The most important factor affecting shift workers was night duty [7,8]. Moreover, shift nurses having to work irregular shifts or three shifts (morning-evening-night shifts) had worse sleep quality than nurses working continuous shifts [6,9]. Poor sleep may negatively impact physical health by promoting unhealthy eating habits, obesity, metabolic syndrome, and diabetes [10,11,12]. Unusual working hours leads to behavior changes such as an altered diet and weight gain, resulting in inadequate sleep [13,14].

Childhood trauma, such as physical, sexual, or emotional abuse and emotional or physical neglect, are associated with sleep disturbances [15,16]. Compared to those without a history of abuse, adults who have experienced sexual abuse in childhood tend to have poor sleep quality, and those who have experienced emotional and physical abuse often have trouble falling or staying asleep [15,17,18]. Evidence suggests that adults who have experienced childhood traumatic events are at high risk for sleep disorders. Additionally, severe childhood adverse events, including abuse and neglect, negatively impact mental health and increase stress in adults [19]. However, few studies have evaluated the association between childhood trauma and sleep quality in hospital nurses.

A history of childhood trauma is one of the most common factors associated with eating disorders during adulthood [20,21]. Eating disorders are defined as abnormal eating habits, including consuming unusually large amounts of food or a lack of appropriate food intake [22]. Psychological factors (i.e., body image disturbances and personality traits) and sociocultural factors elevate the risk of eating disorders and might delay full recovery in eating disorder patients [20,23,24]. Additionally, eating disorders (i.e., anorexia nervosa, binge eating disorder, and bulimia nervosa) are more likely to occur in women [25]. However, no research has explored the relationship between childhood trauma and eating disorders in hospital nurses in South Korea.

Another connection between poor eating habits and sleep hygiene could be circadian de-synchronization, which is experienced by people who suffer from eating disorders [26,27]. Eating disorders are highly prevalent among female Korean nurses [28]. Korean nurses have worse sleep quality compared to those who work in other countries [1,29,30]. However, to date, few studies have examined the relationship between eating disorders and sleep quality among Korean shift nurses.

Nurses play a significant role in the 24 h care of hospitalized patients. However, little is known about how nurses’ childhood maltreatment affects their quality of care and sleep quality [19,31,32]. Childhood trauma in women is of particular interest, because approximately 91% of registered nurses in South Korea are female, and women are at increased risk for sexual, emotional, and physical abuse [33,34], which are associated with insufficient sleep quality (i.e., difficulty falling asleep and nocturnal awakening) [35]. Therefore, this study aims to determine the relationship between childhood trauma, eating disorders, and sleep quality in hospital nurses and to identify the factors that affect sleep quality in hospital nurses.

## 2. Materials and Methods

### 2.1. Study Design and Sample

The study has a descriptive, cross-sectional design. A self-reported questionnaire examined the relationship between childhood trauma, eating disorders, and sleep quality among registered hospital nurses in South Korea.

A convenience sample of hospital nurses was recruited from six general hospitals in Jeonbuk, an urban area in South Korea. The inclusion criteria were registered hospital nurses who work in three shifts (morning, evening, and night shifts) in hospitals and had more than six months of clinical experience. We excluded nurses that were pregnant, who were using sleep drugs or other pharmacological treatments, or who had no clinical experience, such as those in training or who had worked less than six months. The principal investigator (PI) contacted the directors of seven general hospitals (>200 beds) to obtain permission for recruitment. To determine the sample size, we performed a power analysis using G Power 3.1.2 [36] for multivariate regression analysis. The desired sample size to compute a test power (1-β) of 0.95 with 16 predictors was 204, with an effect size of 0.15 and an alpha of 0.05. Among the 290 total participants in the study, respondents whose random responses were detected or who did not complete the pre-specified variables were excluded from the study. After excluding those with missing values, a total of 279 eligible participants were included in the study, a response rate of approximately 96.2%.

### 2.2. Measures

Demographic characteristics included age, marital status, economic status, religion, alcohol consumption, smoking, chronic disease, and body mass index (BMI). Instruments for this study were used after obtaining approval from the authors.

#### 2.2.1. Sleep Quality

Sleep quality was assessed using the Korean version of the Pittsburgh Sleep Quality Index (PSQI-K). This tool was translated by Sohn et al. [37] based on the original version [28], and its reliability and validity were verified. The PSQI-K reflects the sleep quality of an individual over the past month. This questionnaire consists of 19 self-report items and measures seven components: subjective sleep quality, sleep duration, latency, habitual sleep efficiency, sleep disturbances, use of sleep medication, and daytime dysfunction. Each item was scored on a four-point Likert scale, ranging from “never” (0) to “over three times a week” (3). The global PSQI-K scores were summed to obtain a total score ranging from 0 to 21, with a higher score indicating worse sleep quality. Based on the criteria set by Buysse et al. [38], we categorized participants with PSQI-K scores of ≤5 as having good sleep quality and those with a score of >5 as having poor sleep quality.

#### 2.2.2. Eating Disorders

Eating disorders were measured with a tool that assesses binge-eating behavior in adults, a 15-item self-report questionnaire used by Jeong [39]. It was reviewed, modified, and re-deemed based on the Eating Disorder Inventory-1, Bulimia Test-Revised, and the Korean Eating Attitude Test-26. Previous research demonstrated good levels of validity and reliability [39]. The tools are based on eating disorders experienced in the past three months. Items are scored on a five-point Likert scale from “not at all” (1) to “almost always” (5), with a higher score indicating more frequent eating disorder events.

#### 2.2.3. Childhood Trauma

Childhood trauma was assessed using the Korean version of the Childhood Trauma Questionnaire (CTQ). This tool was translated by Yu et al. [40] based on the original version [41], and its reliability and validity were verified. This questionnaire consists of 28 self-report items and assesses five types of trauma experienced as a child or teenager (emotional abuse, physical abuse, sexual abuse, emotional neglect, and physical neglect) and three items about the validity scale (minimization/denial scale). Each item is scored on a five-point Likert scale from “never true” (1) to “very often true” (5). These scores were summed to yield a total score for each trauma ranging from 5 to 25, with higher scores indicating greater trauma severity.

#### 2.2.4. Data Collection

Data were collected by a structured questionnaire from 1 June 2020, to 5 July 2020. We explained the purpose of the study to the directors of the nursing departments of six general hospitals and requested approval to perform the study and collect data. Researchers visited the nursing departments of each hospital directly and provided each participant with a questionnaire contained in an individual envelope. Before data collection, all participants were assured that survey participation would not influence them in any way and that the survey data would be discarded directly after coding for research analysis. All participants provided written consent before enrollment. To protect the respondents’ privacy, the completed questionnaire contained no individual identifiers and was sealed in an envelope before it was collected by the researcher. All participants were compensated with small gifts.

#### 2.2.5. Ethical Considerations

The study protocol was approved by the relevant institutional review board (IRB Number. 1040647-202004-HR-002-02). All participants were required to provide informed consent in compliance with IRB regulations. Participants could voluntarily withdraw their participation at any time.

#### 2.2.6. Data Analysis

Data were analyzed using IBM SPSS Statistics, version WIN 25.0 (IBM Corp., Armonk, NY, USA). The participants’ general and clinical characteristics, eating disorders, childhood trauma, and sleep quality were analyzed with descriptive statistics. Differences in sleep quality according to participant characteristics were tested with *t*-tests, ANOVA, and Scheffé post hoc tests. The correlation between continuous variables was analyzed using Pearson correlation analysis. Factors affecting sleep quality were analyzed with a linear regression analysis. The statistical significance level was set at *p* < 0.05.

## 3. Results

### 3.1. Subjects’ Demographic Characteristics

A total of 290 participants were included in this study, 279 of whom completed the survey questionnaire (a response rate of 96.2%). As shown in Table 1, the mean age of the participants was 36.9 years (ranging from 23–60 years) and 62% of subjects were 20–39 years old.

All participants were female. A majority of the participants reported being married (55.6%) and over half reported their economic status as higher than middle class (87.1%). The majority of participants reported having no religious affiliation (44.4%), not smoking (97.1%), and consuming alcohol (61.6%). Of these, 26.9% consumed alcohol less than once a month, 28.0% consumed alcohol two to four times a month, and 6.8% consumed two or more servings of alcohol per week. Their average BMI was 21.3 kg/m^2^ (SD = 5.4); 83.5% (*n* = 233) had a normal BMI and 14.3% (*n* = 40) were overweight. The percentage of respondents reporting good and poor sleep quality was 67.0% and 33.0%, respectively.

### 3.2. Levels of Variables and Correlations between Variables

Table 2 presents the levels of eating disorders, childhood trauma, and sleep quality. The total PSQI scores ranged from 0 to 19 (M = 6.1, SD = 3.7). The mean PSQI score for good sleep quality was 2.1 (SD = 1.4), and for poor sleep quality was 8.1 (SD = 2.8). Table 2 shows more information on the PSQI. The mean score for eating disorders was 39.6 (SD = 13. 5). The mean CTQ score was 52.9 (SD = 10.3), followed by physical abuse (M = 6.7, SD = 3.1), emotional abuse (M = 6.0, SD = 2.4), sexual abuse (M = 6.5, SD = 2.1), physical neglect (M = 9.0, SD = 3.1), and emotional neglect (M = 9.6, SD = 4.7).

Table 3 presents the correlations between study variables, which reveals that sleep quality was significantly and positively correlated with physical (r = 0.205, *p* = 0.001) and emotional abuse (r = 0.236, *p* < 0.001). Eating disorders were significantly and positively correlated with emotional abuse (r = 0.151, *p* = 0.012) and neglect (r = 0.188, *p* = 0.002).

### 3.3. Factors Influencing Sleep Quality

Table 4 shows the factors that influence sleep quality. The most significant factor was alcohol consumption (*β* = 0.267, *p* < 0.001), followed by emotional abuse (*β* = 0.188, *p* = 0.01), age (*β* = −0.154, *p* = 0.10), chronic disease (*β* = −0.124, *p* = 0.28), and BMI (*β* = 0.110, *p* = 0.048). These factors explained 17% of the total variance in sleep quality (F = 12.369, *p* < 0.001, R^2^ = 0.185, Adj. R^2^ = 0.170).

## 4. Discussion

This study analyzed the correlations between childhood trauma, eating disorders, and sleep quality and identified predictors of sleep quality. To that end, five variables were found to be significantly associated with sleep quality: age, alcohol consumption, chronic disease, BMI, and emotional abuse.

In this study, 61.6% of respondents reported consuming alcohol. Of these, 6.8% consumed two or more servings of alcohol per week. Previous research [42] has reported that of the 78% of respondents who drank alcohol, 25% were at risk of alcohol use disorders, and used alcohol as a sleeping aid. Future research should investigate the amount of alcohol consumed by nurses, when it is consumed, the type of alcohol consumed and its relation to sleep quality. Approximately 85% of the participants had a normal BMI. This result is not consistent with previous research findings [10,12,43,44]. The mean BMI score of nurses working in Midwestern United States hospitals was 27.8 [10], and in public hospitals in Brazil was 26.1 [12]. Nearly 49% of nurses in a community hospital in New York state [43] and 69% of healthcare professionals at Belagavi hospital in India had a normal BMI [44]. This score may differ depending on factors such as region, race, gender, and socioeconomic status [10,12,43,44].

The mean eating disorder scores were 39.5, higher than the 34.6 obtained in the previous study [39]. Nurses had more job strains compared to other occupations. Job strains tend to encourage consuming food from canteens and snacking [12,44], and may lead to unhealthy eating habits, including an irregular amount of meals and an irregular total number of eating events per day [12]. As a result, eating disorders may increase the number of nurses overweight and obese, which can affect a nurse’s job performance [43]. The mean CTQ score (followed by physical abuse, emotional abuse, sexual abuse, physical neglect, and emotional neglect) was 52.9 in this study, which is supported by previous research findings [15,24]. According to previous research, 63.9% of adults have experienced at least one adverse childhood experience [24]. Adverse childhood experiences not only result in a negative self-evaluation but also a reduced capacity to self-comfort, resulting in a restricted self-development [19]. Nurses who have experienced childhood trauma that have problems with self-worth, trust, and assertiveness may find that it affects their practice and relationships with others [31]. Therefore, future studies should investigate nurses’ childhood trauma experiences and their effect factors.

The mean PSQI scores of 6.1 in this study is consistent with the score of 6.8 in previous research [1]. We found that poor sleep quality was reported in nearly 33.0% of respondents, which is in line with the 32.6% of people who had trouble falling or staying asleep in previous research [17]. Another study [45] found that 28.7% of males and 42.5% of females were bad sleepers, which was nearly identical to our study. According to previous research, people who reported trouble falling asleep or staying asleep were also four times more likely to report feeling tired, even after a good night’s sleep [17]. For this reason, sleep deprivation also leads to irritability, a bad mood, reduced communication skills, and an inability to cope with the emotional demands of the workplace [1,2]. This can impair a nurse’s ability to respond to patient needs and patient safety. To improve nurses’ sleep quality and improve their knowledge about maintaining a healthy lifestyle, health care institutions will have to plan and operate workplace health promotion programs [10].

In this study, the factors influencing sleep quality included age, alcohol consumption, chronic disease, BMI, and emotional abuse in the CTQ. Age was affecting the sleep quality of hospital nurses, which is consistent with previous research findings [17]. This result is probably because the evolutive changes that occur throughout adult life impact the sleep rhythm regulation system [46]. Most female nurses experience menopause and its accompanying hormonal changes as they age. As a previous study [46] suggests, this may be related to sleep quality. However, this study did not investigate whether menopause can affect sleep quality. The effect of BMI and chronic disease were also consistent with previous research findings [4,10]. Hospital nurses develop risk factors, such as unhealthy sleep quality and eating habits, due to their adaptations to their work routines [47]. As a result, nurses are at an increased risk of obesity, which may lead to chronic disease [3,12]. This may be linked to indirect costs, such as absenteeism, skipping work, and reduced work productivity [10].

Eating disorders among female Korean nurses were highly prevalent [28]. Korean nurses have worse sleep quality compared to those who work in other regions, including China and Europe [1,29,30]. However, eating disorders were not related to sleep quality in this study, although previous research has reported that they affect the quality of life by reducing sleep quality [24]. Eating itself, rather than gaining weight or being obese, may cause sleep deficits [43]. Although we did not explore this concept, eating is associated with an increase in diabetes, cardiovascular disease, and obesity [3]. Therefore, future studies should investigate the relationship between eating disorders and adverse health outcomes that affect the quality of sleep in hospital nurses.

We found that emotional abuse in childhood trauma affected sleep quality, which supports previous research findings [15,35]. Emotional abuse in childhood trauma has a cumulative negative impact on sleep physiology and underlies the physiological and psychosocial health problems of nurses [17,35,48]. Adverse childhood experiences may interfere with coping strategies to handle adversity and may lead to higher stress levels over time [15,48]. It also may increase the risk of eating disorders, such as binge eating and fasting/skipping meals, and increase the risk of primary insomnia [15,24]. Therefore, healthcare workers, including nurses, need to be aware of the childhood trauma experiences that threaten their sleep health. The poor sleep quality of hospital nurses can threaten the quality of care and patient safety.

Our study is the first to examine the relationship between childhood trauma, eating disorders, and sleep quality in hospital nurses. In particular, we were proven that childhood trauma can affect the quality of sleep of nurses of hospital nurses. However, this study has some limitations. Firstly, due to the cross-sectional design, no causal relationships can be derived from the conclusions. Studies with longitudinal and experimental research designs are needed to confirm the causality tested in the study. Secondly, this study used a convenience sample, from one urban area of South Korea. The results of this study might not be generalizable to all South Korean clinical nurses. Our results are also susceptible to selection bias. Future research should be conducted on a more diverse group of hospital nurses and should incorporate more detailed subjective sleep difficulty measures and objective sleep data. Thirdly, these results should be interpreted carefully, as the possibility of recall bias may have influenced retrospective reports of childhood trauma. Finally, this study did not account for menopause, which is related to sleep quality. Future study should consider these variables. It should be noted that the data were collected during the COVID-19 pandemic.

## 5. Conclusions

Hospital nurses need to increase their knowledge regarding sleep health to maintain a healthy lifestyle while working as a nurse. Therefore, hospital and nursing managers should consider strategies to prevent and intervene in the sleep quality threats attributed to the adverse childhood experiences of hospital nurses. Hospital managers should develop workplace health programs to increase awareness of sleep health, establish a flexible, rotating work schedule, and enact social support policies. These strategies will provide more suitable living and working conditions for nurses and reduce their risk factors for chronic diseases.

## Figures and Tables

**Table 1:** Statistics describing the Study Population (*n* = 279).

Variable	*n* (%)
Mean age (years) (SD, range)	36.9 (9.9) (23 to 60)
20–39	173 (62.0)
40–49	64 (22.9)
≥50	42 (15.1)
Marital status	
Married	155 (55.6)
Single/divorced	124 (44.4)
Economic status	
≥Middle	243 (87.1)
Low	36 (12.9)
Religion	
Christianity	95 (34.1)
Roman Catholicism	39 (14.0)
Buddhism	21 (7.5)
None	124 (44.4)
Alcohol consumption (frequency)	
Never	107 (38.4)
Once a month	75 (26.9)
Two to four times a month	78 (28.0)
Two or more times a week	19 (6.8)
Smoking	
No	271 (97.1)
Yes	8 (2.9)
Chronic disease	
Yes	42 (15.1)
No	237 (84.9)
BMI (kg/m^2^)	21.3 (5.4)
Normal weight (18.5 to 24.9)	233 (83.5)
Overweight (25 to 29.9)	40 (14.3)
Obesity (30 or greater)	6 (2.2)
Sleep quality	
Good sleep quality (PSQI ≤ 5)	187 (67.0)
Poor sleep quality (PSQI > 5)	92 (33.0)

Note: BMI, Body Mass Index; SD, Standard Deviation.

**Table 2:** Sleep Quality, Eating Disorders, and Childhood Trauma Levels (*n* = 279).

Variables	Min–Max	Mean (SD)
Total PSQI score	0–19	6.1 (3.7)
Subjective sleep quality	0–3	1.1 (0.6)
Sleep latency	0–3	1.8 (1.3)
Sleep duration	0–3	0.6 (0.9)
Habitual sleep efficiency	0–3	0.5 (0.9)
Sleep disturbances	0–3	0.9 (0.6)
Use of sleeping medicine	0–3	0.1 (0.5)
Daytime dysfunction	0–3	0.9 (0.8)
Good sleep quality		2.1 (1.4)
Poor sleep quality		8.1 (2.8)
Eating disorders	17–81	39.5 (13.5)
CTQ Total	29–85	52.9 (10.3)
Physical abuse	5–25	6.7 (3.1)
Emotional abuse	4–19	6.0 (2.4)
Sexual abuse	5–26	6.5 (2.1)
Physical neglect	5–18	9.0 (3.1)
Emotional neglect	5–25	9.6 (4.7)

Note: SD, Standard Deviation; CTQ, Childhood Trauma Questionnaire; PSQI, Pittsburgh Sleep Quality Index.

**Table 3:** Correlations of Study Variables (*n* = 279).

Variable	Sleep Quality	Eating Disorders	Physical Abuse	Emotional Abuse	Sexual Abuse	Physical Neglect	Emotional Neglect
Sleep quality	-		.				
Eating disorders	0.053 (0.375)	-					
Physical abuse	0.205 (0.001)	0.084 (0.164)	-				
Emotional abuse	0.236 (<0.001)	0.151 (0.012)	0.406 (< 0.001)	-			
Sexual abuse	0.088 (0.143)	0.049 (0.420)	0.200 (0.001)	0.267 (< 0.001)	-		
Physical neglect	−0.071 (00.237)	−0.058 (0.330)	0.106 (0.076)	0.032 (0.596)	0.175 (0.003)	-	
Emotional neglect	0.040 (0.510)	0.188 (0.002)	0.199 (0.001)	0.420 (< 0.001)	0.123 (0.040)	0.067 (0.265)	-

**Table 4:** Factors Affecting Sleep Quality (*n* = 279).

Variables	B	SE	β	t	*p*	*R^2^*	*Adj. R^2^*	*F*	*p*
Constant	5.784	1.884		3.070	0.002	0.185	0.170	12.369	<0.001
Age	−0.058	0.022	−0.154	−2.596	0.010				
Alcohol consumption	1.032	0.233	0.267	4.636	<0.001				
Chronic disease	−1.294	0.586	−0.124	−2.208	0.028				
BMI	0.929	0.467	0.110	1.989	0.048				
Emotional abuse	0.285	0.085	0.188	3.341	0.001				

Note: BMI, Body Mass Index; SE, Standard error.

## References

[1] Gómez-García T., Ruzafa-Martínez M., Fuentelsaz-Gallego C., Madrid J.A., Rol M.A., Martínez-Madrid M.J., Moreno-Casbas M.T. (2016). Nurses’ sleep quality, work environment and quality of care in the Spanish National Health System: Observational study among different shifts. BMJ Open.

[2] Deng X., Liu X., Fang R. (2020). Evaluation of the correlation between job stress and sleep quality in community nurses. Medicine.

[3] Itani O., Jike M., Watanabe N., Kaneita Y. (2017). Short sleep duration and health outcomes: A systematic review, meta-analysis, and meta-regression. Sleep Med..

[4] Korompeli A., Muurlink O., Tzavara C., Velonakis E., Lemonidou C., Sourtzi P. (2014). Influence of Shiftwork on Greek Nursing Personnel. Saf. Health Work.

[5] Yoo G.S., Kim T.W. (2017). The Effect of Morningness-Eveningness on Shift Work Nurses: Sleep Quality, Depressive Symptoms and Occupational Stress. Sleep Med. Res..

[6] Zencirci A.D., Arslan S. (2011). Morning-evening type and burnout level as factors influencing sleep quality of shift nurses: A questionnaire study. Croat. Med J..

[7] Cè E., Doria C., Roveda E., Montaruli A., Galasso L., Castelli L., Mulè A., Longo S., Coratella G., D’Aloia P. (2020). Reduced Neuromuscular Performance in Night Shift Orthopedic Nurses: New Insights From a Combined Electromyographic and Force Signals Approach. Front. Physiol..

[8] Shao M.-F., Chou Y.-C., Yeh M.-Y., Tzeng W.-C. (2010). Sleep quality and quality of life in female shift-working nurses. J. Adv. Nurs..

[9] Chang W.-P., Chang Y.-P. (2019). Relationship between job satisfaction and sleep quality of female shift-working nurses: Using shift type as moderator variable. Ind. Health.

[10] Beebe D., Chang J.J., Kress K., Mattfeldt-Beman M. (2017). Diet quality and sleep quality among day and night shift nurses. J. Nurs. Manag..

[11] Zhao I., Turner C. (2008). The impact of shift work on people’s daily health habits and adverse health outcomes. Aust. J. Adv. Nurs..

[12] Griep R.H., Bastos L.S., Fonseca M.D.J.M.D., Silva-Costa A., Portela L.F., Toivanen S., Rotenberg L. (2014). Years worked at night and body mass index among registered nurses from eighteen public hospitals in Rio de Janeiro, Brazil. BMC Health Serv. Res..

[13] Berkey C.S., Rockett H.R., Colditz G. (2008). Weight Gain in Older Adolescent Females: The Internet, Sleep, Coffee, and Alcohol. J. Pediatr..

[14] Knutsson A. (2003). Health disorders of shift workers. Occup. Med..

[15] Bader K., Schäfer V., Schenkel M., Nissen L., Schwander J. (2007). Adverse childhood experiences associated with sleep in primary insomnia. J. Sleep Res..

[16] Gregory A.M., Caspi A., Moffitt T.E., Poulton R. (2006). Family Conflict in Childhood: A Predictor of Later Insomnia. Sleep.

[17] Chapman D.P., Wheaton A.G., Anda R.F., Croft J.B., Edwards V.J., Liu Y., Sturgis S.L., Perry G.S. (2011). Adverse childhood experiences and sleep disturbances in adults. Sleep Med..

[18] Gal G., Levav I., Gross R. (2011). Psychopathology among adults abused during childhood or adolescence: Results from the Israel-based World Mental Health Survey. J. Nerv. Ment. Dis..

[19] Maunder R.G., Peladeau N., Savage D., Lancee W.J. (2010). The prevalence of childhood adversity among healthcare workers and its relationship to adult life events, distress and impairment. Child Abus. Negl..

[20] Caslini M., Bartoli F., Crocamo C., Dakanalis A., Clerici M., Carrà G. (2016). Disentangling the association between child abuse and eating disorders: A systematic review and meta-analysis. Psychosom. Med..

[21] Groth T., Hilsenroth M., Boccio D., Gold J. (2019). Relationship between Trauma History and Eating Disorders in Adolescents. J. Child Adolesc. Trauma.

[22] Sharan P., Sundar A.S. (2015). Eating disorders in women. Indian J. Psychiatry.

[23] A Rikani A., Choudhry Z., Choudhry A.M., Ikram H., Asghar M.W., Kajal D., Waheed A., Mobassarah N.J. (2013). A critique of the literature on etiology of eating disorders. Ann. Neurosci..

[24] Hazzard V.M., Bauer K.W., Mukherjee B., Miller A.L., Sonneville K.R. (2019). Associations between childhood maltreatment latent classes and eating disorder symptoms in a nationally representative sample of young adults in the United States. Child Abus. Negl..

[25] Kass A.E., Trockel M., Weisman H., Fitzsimmons-Craft E.E., Balantekin K.N., Wilfley D.E., Taylor C.B. (2018). A screening tool for detecting eating disorder risk and diagnostic symptoms among college-age women. J. Am. Coll. Health.

[26] Roveda E., Montaruli A., Galasso L., Pesenti C., Bruno E., Pasanisi P., Cortellini M., Rampichini S., Erzegovesi S., Caumo A. (2017). Rest-activity circadian rhythm and sleep quality in patients with binge eating disorder. Chrono Int..

[27] Verster J.C., Tromp M.D., Donners A.A., Garssen J. (2016). Sleep, eating disorder symptoms, and daytime functioning. Nat. Sci. Sleep.

[28] Kim O., Kim M.S., Kim J., Lee J.E., Jung H. (2018). Binge eating disorder and depressive symptoms among females of child-bearing age: The Korea Nurses’ Health Study. BMC Psychiatry.

[29] Hong K.J., Lee Y. (2020). The Moderating Effect of Nursing Practice Environment on the Relationship between Clinical Nurses’ Sleep Quality and Wellness. Int. J. Environ. Res. Public Health.

[30] Zhang L., Sun D.-M., Li C.-B., Tao M. (2016). Influencing Factors for Sleep Quality Among Shift-working Nurses: A Cross-Sectional Study in China using 3-factor Pittsburgh Sleep Quality Index. Asian Nurs. Res..

[31] Díaz-Olavarrieta C. (2001). Prevalence of Intimate Partner Abuse among Nurses and Nurses’ Aides in Mexico. Arch. Med. Res..

[32] Gallop R., McKeever P., Toner B., Lancee W., Lueck M. (1995). The impact of childhood sexual abuse on the psychological well-being and practice of nurses. Arch. Psychiatr. Nurs..

[33] Finkelhor D., Turner H.A., Shattuck A., Hamby S.L. (2013). Violence, crime, and abuse exposure in a national sample of children and youth: An update. JAMA Pediatr..

[34] Kim S.H. (2013). Over 1000 male students have passed the national nursing exam this year. Korean Nurses Assoc. News.

[35] Kajeepeta S., Gelaye B., Jackson C.L., Williams M.A. (2015). Adverse childhood experiences are associated with adult sleep disorders: A systematic review. Sleep Med..

[36] Faul F., Erdfelder E., Lang A.-G., Buchner A. (2007). G*Power 3: A flexible statistical power analysis program for the social, behavioral, and biomedical sciences. Behav. Res. Methods.

[37] Sohn S.I., Kim D.H., Lee M.Y., Cho Y.W. (2012). The reliability and validity of the Korean version of the Pittsburgh Sleep Quality Index. Sleep Breath..

[38] Buysse D.J., Reynolds C.F., Monk T.H., Berman S.R., Kupfer D.J. (1989). The Pittsburgh sleep quality index: A new instrument for psychiatric practice and research. Psychiatry Res..

[39] Jeong S.J. (2010). The Effect of Impulsiveness and Social Support on Binge Eating and Drinking Problem: The Mediation of Difficulties in Emotional Regulation. Master’s Thesis.

[40] Yu J.H., Park J.S., Park D.H., Ryu S.H., Ha J.H. (2009). Validation of the Korean childhood trauma questionnaire: The practical use in counseling and therapeutic intervention. Korean J. Health Psychol..

[41] Berstein D., Fink L. (1998). Childhood Trauma Questionnaire: A Retrospective Self-Report.

[42] Machado A., Silva D., Monteiro P.S., Ribeiro L.M., Guilhem D. (2016). Alcohol use by nurses and its effects on health care-Integrative review. Cogitare Enferm..

[43] Ku B., Phillips K.E., Fitzpatrick J.J. (2019). The relationship of body mass index (BMI) to job performance, absenteeism and risk of eating disorder among hospital-based nurses. Appl. Nurs. Res..

[44] Shinde V.V. (2019). Relationship of body mass index to job stress and eating behaviour in health care professionals-an observational study. Obes. Med..

[45] Hinz A., Glaesmer H., Brähler E., Löffler M., Engel C., Enzenbach C., Hegerl U., Sander C. (2017). Sleep quality in the general population: Psychometric properties of the Pittsburgh Sleep Quality Index, derived from a German community sample of 9284 people. Sleep Med..

[46] Madrid-Valero J.J., Martínez-Selva J.M., Couto B.R.D., Sánchez-Romera J.F., Ordoñana J.R. (2017). Age and gender effects on the prevalence of poor sleep quality in the adult population. Gac. Sanit..

[47] Grillo L., Albuquerque N., Vieira N., Mezadri T., Lacerda L. (2018). Risk and protective factors for the development of chronic diseases in nurses. Rev. Enferm. Ref..

[48] Brindle R.C., Cribbet M.R., Samuelsson L.B., Gao C., Frank E., Krafty R.T., Thayer J.F., Buysse D.J., Hall M.H. (2018). The Relationship between Childhood Trauma and Poor Sleep Health in Adulthood. Psychosom. Med..

